# New evidence for the antiquity of *Desmostylus* (Desmostylia) from the Skooner Gulch Formation of California

**DOI:** 10.1098/rsos.221648

**Published:** 2023-06-14

**Authors:** Kumiko Matsui, Nicholas D. Pyenson

**Affiliations:** ^1^ Department of Paleobiology, National Museum of Natural History, Smithsonian Institution, Washington, DC, USA; ^2^ Kyushu University Museum, Kyushu University, Fukuoka, Japan; ^3^ Department of Paleontology and Geology, Burke Museum of Natural History and Culture, Seattle, WA, USA

**Keywords:** fossil, marine mammal, teeth, neogene, paleogene

## Abstract

*Desmostylus* is an extinct marine mammal genus that belongs to Desmostylia, a clade of extinct herbivorous mammals. While desmostylian remains are widely reported from Paleogene and Neogene marine strata of the North Pacific Rim, occurrences of the genus *Desmostylus* are almost entirely limited to middle Miocene strata, with only a few early Miocene records from Japan. Here we report a *Desmostylus* tooth from the earliest Miocene (Aquitanian) Skooner Gulch Formation in northern California, USA. This specimen exhibits cuspules around the crown, a primitive trait of the subfamily Desmostylidae, as seen in more basal branching desmostylid taxa such as *Cornwallius* and *Ounalashkastylus*, but with a high tooth crown and thickened enamel. The specimen is also diagnostically different from all other desmostylid genera, such as *Cornwallius,* and *Ounalashklastylus*. The Aquitanian age of the Skooner Gulch Formation implies that the distinctive tooth morphology of *Desmostylus* has persisted, largely unchanged, for more than 15 million years and that desmostylids possibly originated in western North America.

## 1. Introduction

Desmostylia were herbivorous marine mammals that have been enigmatic since their discovery because of their unusual skeletal and dental morphologies, as well as their unresolved relationships with other mammals, including potentially Perissodactyla (Eurasiatheria) or Tethytheria (Afrotheria) (e.g. [1–3]). Their fossil remains have been found in Oligocene and Miocene marine strata across the North Pacific Rim (e.g. [4–6]). Two major clades within Desmostylia include Paleoparadoxiidae [3] and Desmostylidae, the latter uniquely characterized by high tooth crowns with bundled columnar teeth (e.g. [5]). Throughout desmostylid evolution, tooth crown increases in height, yet tooth enamel remains consistent in thickness, from basal branching desmostlyid lineages such as *Ashoroa* to later diverging species of *Desmostylus* (e.g. [5,7]). There are numerous fossils of *Desmostylus* from the middle Miocene of California (e.g. Temblor Formation [4], see age [8]). However, before the middle Miocene, the fossil record of this genus is sparse (e.g. [9,10]).

Among these occurrence data, there are two sets of occurrences that rank among the oldest records for the genus *Desmostylus* (table 1): three specimens were mentioned (but undescribed) from the Nye Formation of Lincoln County, Oregon, and referred to this genus [5, table 4]; and two specimens referred to *Desmostylus* from the Painted Rock Sandstone Member of the Vaqueros Formation in the Cuyama Valley area of San Barbara County, California [21, 5–6], which is likely early Miocene in age (but see below; table 1). Unfortunately, both sets of occurrences are problematic: Inuzuka [5] provided no morphological diagnosis nor description of the Nye specimens, which may be Aquitanian or Chattian in age (see [22,23]) with an age range between 27.4 and 20.7 Ma [12]; and while Mitchell & Repenning [11] provisionally assigned two fragmentary teeth from the Cuyama Valley to the genus *Desmostylus*, these specimens are smaller and too incomplete to compare with type specimens of the genus, making it difficult to differentiate the Cuyama Valley material from other potential desmostylid genera such as *Cornwallius* or *Ounalashklastylus*. Aside from these two sets of occurrences, the oldest diagnostic records of *Desmostylus* outside of the USA include a partial mandible with some teeth from the Kameno-o Formation [13], an associated but fragmentary set of upper dentition from the Goyasu Formations of Japan [24], and some molars from the early Miocene marine deposits from South Sakhalin, Russia [13,24,25] although the locality and horizons for the latter specimens are not well understood. Both Japanese records are Burdigalian, making them younger than the oldest *Desmostylus* records from the USA.

**Table 1.** *Desmostylus* occurrences in the early Miocene.

specimen	formation	morphology	published taxonomy	locality	age	reference
northeast Pacific						
USNM PAL 22922	Painted Sandstone Member of the ‘Vaqueros’ Formation	incomplete molar	*Desmostylus* cf. *D. hesperus*	USGS vertebrate locality M1028, Caliente Range, San Luis Obispo County, California, USA	Aquitanian	[11]
USNM PAL 22923	Painted Sandstone Member of the ‘Vaqueros’ Formation	incomplete molar	*Desmostylus* cf. *D. hesperus*	USGS vertebrate locality M1028, Caliente Range, San Luis Obispo County, California, USA	Aquitanian	[11]
USNM PAL 181744	Nye Mudstone	skull	*Desmostylus japonicus*	north of Lost Creek, south of Newport, Lincoln County, Oregon, USA	Chattian–Burdigalian, 27.4–20.7 Ma	[5,12]
USNM PAL 187310	Nye Mudstone	skull	*Desmostylus japonicus*	Grant Creek, at the Newport Municipal Airport, Lincoln County, Oregon, USA	Chattian–Burdigalian, 27.4–20.7 Ma	[5,12]
USNM PAL 214741	Nye Mudstone	skull partial, partial lower jaw	*Desmostylus japonicus*	Grant Creek, Lincoln County, Oregon, USA	Chattian–Burdigalian, 27.4–20.7 Ma	[5,12]
northwest Pacific						
GSJ F02071, WUHH IX-8	Kameno-o Formation	upper molar, partial lower jaw	*Desmostylus* sp.	Nagakura coal pit, Iwaki City, Fukushima, Japan	Burdigalian, 17.8 Ma	[13,14]
Iwaki Board of Education	Goyasu Formation	partial skull and partial lower jaw	*Desmostylus* sp.	Gohirakubo, Iwaki City, Fukushima, Japan	Burdigalian, 18.0–17.9 Ma	[11,13]
NMNS PV-5600	Akeyo Formation	incomplete skull and jaw	*Desmostylus japonicus*	Yamanouchi, Mizunami City, Gifu, Japan	Burdigalian, 18 Ma	[15–17]
UHR 07428	Hacchorei Formation, Hongo Group	molar (lost)	*Desmostylus* sp.	Nevelsk, Sakhalin Oblast, Russia	early Miocene, 22–17 Ma	[18,19]
UHR 18467	Upper Dowe Formation, Hongo Group	molar	*Desmostylus* sp.	Chehov, Sakhalin Oblast, Russia	early Miocene, 22–17 Ma	[19,20]
UHR 32378	Aushi Formation, Hongo Group	molar	*Desmostylus* sp.	Chehov, Sakhalin Oblast, Russia	early Miocene, 22–17 Ma	[19,20]

**Figure 1.**
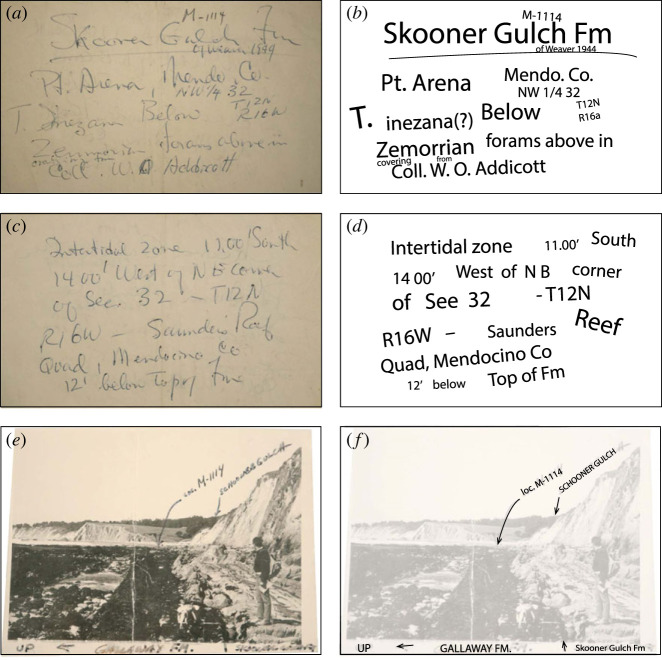
The original labels and photographs that are associated with USNM PAL 706595. (*a*) The front side of the original handwritten label, (*b*) transcribed handwritten text in (*a*). (*c*) The back side of the original handwritten label, (*d*) transcribed handwritten text in (*c*). (*e*) The locality photo attached to the specimen, (*f*) transcribed handwritten text in (*e*).

In the summer of 2021, one of us (K.M.) found an uncatalogued *Desmostylus* tooth in the Paleobiology collections at USNM with handwritten notes and a photograph (figure 1) as the sole associated data. These notes identified Warren O. Addicott (1930–2009) as the source for the specimen, which was collected from the Skooner Gulch Formation, near what is today Schooner Gulch State Park, south of Point Arena, Mendocino County, California. (Note the historical consistency of the spelling of the rock unit versus the geographical locations [25, C2].) Here we describe this single *Desmostylus* tooth, which is among the geologically oldest *Desmostylus* specimens ever described, and its relevance for the evolutionary origin of this genus.

## 2. Material and methods

### 2.1. Institutional abbreviations

AMP, Ashoro Museum of Paleontology, Ashoro, Hokkaido, Japan; B.C.Prov.MUS, Royal British Columbia Museum, Victoria, British Columbia, Canada; GSJ, Geological Museum, National Institute of Advanced Industrial Science and Technology, Japan, Tsukuba, Ibaraki, Japan; MOTA, Museum of the Aleutians, Unalaska, Alaska, USA; NMNS, National Museum of Nature and Science, Tsukuba, Ibaraki, Japan; UCMP, University of California Museum of Paleontology, Berkeley, California, USA; UHR, Hokkaido University Museum, Sapporo, Hokkaido, Japan; USNM PAL, Department of Paleobiology, National Museum of Natural History, Smithsonian Institution, Washington, District of Columbia, USA; WUHH, Honjo High School attached to Waseda University, Honjo, Saitama, Japan; YPM, Yale Peabody Museum, New Haven, Connecticut, USA.

### 2.2. Three-dimensional surface scanning

We used an EinScan-SP, a desktop three-dimensional scanner, and EinScan H, a hand-held structured light scanner (Shining 3D Tech Co. Ltd, Hangzhou, China) to collect three-dimensional surface scan data and then process and create three-dimensional models of teeth from the following Desmostylidae: *Cornwallius sookensis* (USNM PAL 181741), *Ounalashkastylus tomidai* (MOTA 2004.009.04), and *Desmostylus* sp. (USNM PAL 706595). Data cleaning, processing and model creation were completed in EinScan software packages (EXScan S_V3.1.3.0 for EinScan-SP and EXScan H_v1.1.0 for EinScan HI). We converted point clouds from initial captures into watertight three-dimensional models using EinScan processing software. The data underlying this study are available in the electronic supplementary material [26] and at MorphoSource [27]: USNM PAL 181741 (https://doi.org/10.17602/M2/M514948) [28], 706595 (https://doi.org/10.17602/M2/M514928) [29] and MOTA 2004.009.04 (https://doi.org/10.17602/M2/M514952) [30].

## 3. Results

### 3.1. Systematic palaeontology

Desmostylia Reinhart, 1953

Desmostyloidea Osborn, 1905 *sensu* Matsui and Tsuihiji, 2019

Desmostylidae Osborn, 1905 *sensu* Matsui and Tsuihiji, 2019

*Desmostylus* Marsh, 1888

*Desmostylus* sp.

*Diagnosis*. USNM PAL 706595 belongs to Desmostylia, based on the tightly bundled columnar tooth structure. This specimen can be assigned to Desmostylidae based on the length of the tooth columns on the mesial–distal axis, the comparatively high crowns of its teeth, and thickened tooth enamel; in Paleoparadoxiidae, their tooth columns are much lower on the mesial–distal axis, with lower tooth crowns and thinner enamel. The cusps of *Desmostylus* are much larger, higher, and have thicker enamel than *Ashoroa* and *Cornwallius*; in *Ounalashkastylus*, its molars are longer by proportional width and have shorter crown heights.

*Locality*. W. O. Addicott and Richard Pierce collected USNM PAL 706595 within 100 m of 38°51'53.9″ N 123°39'14.7″ W in Mendocino County, California, USA. We infer the precision of this locality by comparing Addicott's [25] description and published photographs with USNM fieldnotes (figure 1; see electronic supplementary material, figure S1). These coordinates are the closest approximation to USGS vertebrate locality M-1114 (see electronic supplementary material, figure S1), which is hand-labelled on a photograph (figure 1) that is penecontemporaneous with a very similar, but not identical one, published by Addicott [25, fig. 3]. This locality is directly south of the topographic low of Schooner Gulch and parallel to the Coastal Highway; Schooner Gulch State Beach and Gallaway Creek are located to the north. This locality is about six kilometers south of Point Arena, Mendocino County, California, USA. USGS vertebrate locality M-114 is likely equivalent to Schooner Gulch 1 (UCMP locality V75135).

*Age*. Upper part of Skooner Gulch Formation, earliest Miocene (Aquitanian) based on benthic foraminiferal stage and geochronological data (see [12,31]). From the original handwritten label in USNM collections (figure 1), we were able to determine that USNM PAL 706595 was collected during geological survey work by staff of the United States Geological Survey (USGS). Along with a rich fossil molluscan assemblage (e.g. *Turritella inezana*, *Chlamys* cf. *C. hertleini*) [25], many fossil marine vertebrates have been identified from the Skooner Gulch Formation exposed on the coastline south of what is now Schooner Gulch State Park [32]. The broader assemblage of fossil marine vertebrates from this formation includes over a dozen elasmobranch taxa (e.g. *Megachasma applegatei*, *Carcharocles auriculatus*) [32,33] and other fossil marine mammals, including early odontocetes (cf. *Argyrocetus* sp.) [32], and the type specimen of *Archaeoparadoxia weltoni* [31], a paleoparadoxiid desmostylian. Recently Poust & Boessenecker [12] described additional material of *Enaliarctos mealsi*, a stem pinniped, from the upper glauconitic sandstones of the Skooner Gulch Formation at this locality, which they inferred is 23.03–22 Ma (Aquitanian, see below). Notably, the horizon bearing the type of *Archaeoparadoxia weltoni* was 5.7 m below the top of the Skooner Gulch Formation and its conformable contact with the overlying Gallaway Formation [31].

In his monographic work, Addicott [25, C4] mentioned that the top of the Skooner Gulch Formation has

…a 15-foot [4.6 m] interval of ingrained glauconitic sandstones with abundant nodules of phosphatic material up to 3 inches [7.6 cm] in diameter. Scattered shark teeth, fish vertebrae, and bone fragments occur in these uppermost fine-grained sandstones. A cheek tooth of *Desmostylus* (identified by C. A. Repenning, oral commun., Oct. 1966) has also been collected from near the top of the formation.

Several pages later, Addicott [25, C9] elaborated that the ‘cheek tooth of *Desmostylus*, collected by the writer and Richard Pierce 12 feet [3.7 m] below the top of the Skooner Gulch Formation (USGS vertebrate locality M1114), is compatible with the molluscan evidence of an early Miocene age for the formation’. This stratigraphic precision indicates that USNM PAL 706595 was collected from a slightly higher level near the top of the Skooner Gulch Formation, about 4 m above the stratigraphic level of the type locality of *Archaeoparadoxia weltoni*.

Recently, Poust & Boessenecker [12] constrained the age of the Skooner Gulch Formation to 23.03–22 Ma through several lines of evidence. First, the Skooner Gulch Formation unconformably overlies the Oligocene Iversen Basalt, which has a K/Ar date of 23.8 Ma [34,35]. Second, because Oligocene microfossils are absent from the Skooner Gulch Formation [36], the entire formation can be constrained to the Miocene, as Barboza *et al*. [37] proposed, based on studies from coeval rock units from southern California. Lastly, Phillips *et al*.'s [32] studies on foraminifera from exposures near Schooner Gulch identified the Zemorrian–Saucesian boundary within the lower part of the overlying Gallaway Formation, with Prothero *et al*. [38] indicating that the Zemorrian benthic foraminiferal stage is as young as 22 Ma, depending on the locality (i.e. Aquitanian; see also [39]). Thus, the Skooner Gulch Formation ranges between 23.03 and 22 Ma.

### 3.2. Description

**Figure 2.**
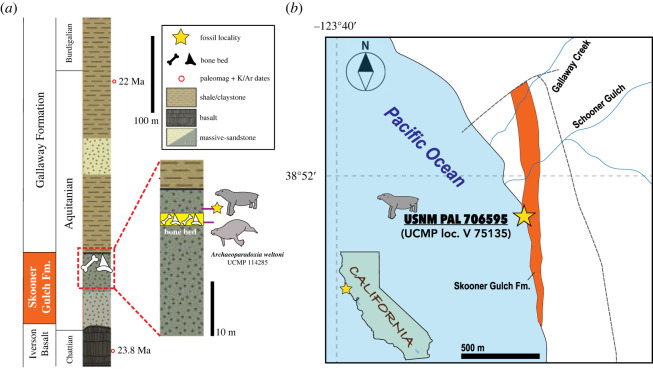
The horizon and locality of USNM PAL 706595. (*a*) Stratigraphic column of Schooner Gulch area, modified from Poust & Boessencker [12] and Phillips *et al*. [32]. (*b*) Locality map for USNM PAL 706595 based on Addicott [25]. Orange indicates the Skooner Gulch Formation; the star denotes the locality of USNM PAL 706595.

**Figure 3.**
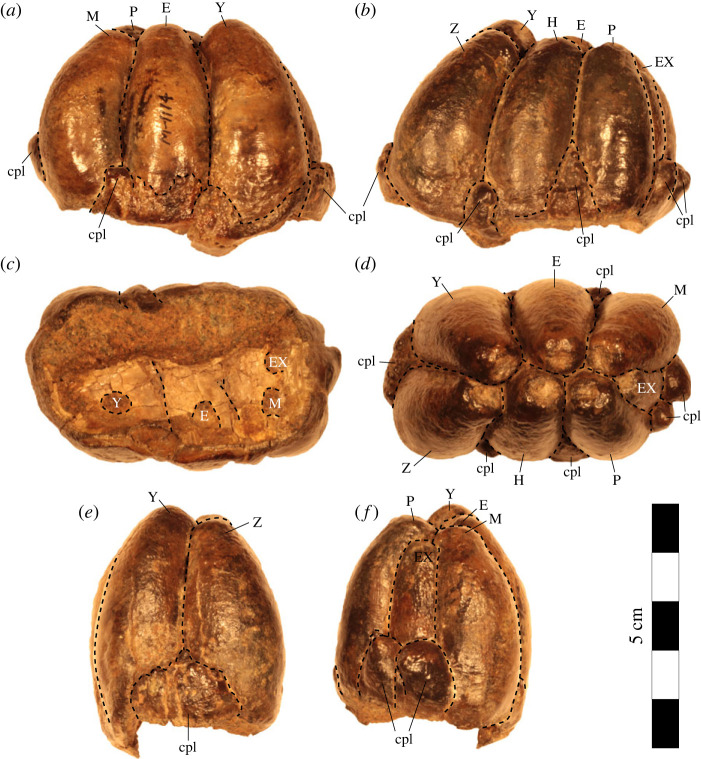
*Desmostylus* sp. (USNM PAL 706595), lower molar (likely m2). Tooth in (*a*) lateral view; (*b*) lingual view; (*c*) ventral view; (*d*) occlusal view; (*e*) anterior and (*f*) posterior views. Abbreviations: cpl, cuspule; E, entoconid; EX, extra cusp; H, hypoconid; M, metaconid; P, protoconid; Y and Z, distal talonid (*sensu* Inuzuka *et al*. [5]).

**Figure 4.**
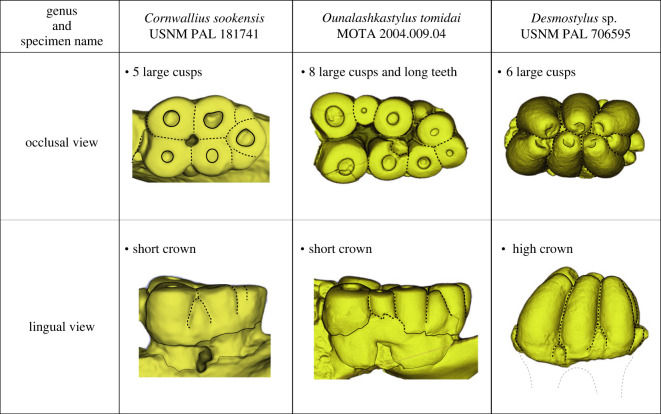
Tooth morphological comparisons among Desmostylidae including *Cornwallius sookensis* (USNM PAL 181741), *Ounalashkastylus tomidai* (MOTA 2004.009.04), and *Desmostylus* sp. (USNM PAL 706595). Three-dimensional models were uploaded to MorphoSource, specifically USNM PAL 181741 (https://doi.org/10.17602/M2/M514948), 706595 (https://doi.org/10.17602/M2/M514928), and MOTA 2004.009.04 (https://doi.org/10.17602/M2/M514952).

USNM PAL 706595 includes only the left molar crown with seven major cusps with many cuspules. USNM PAL 706595 has typical desmostylodonty (*sensu* Clark [31]). With major cusps arranged vertically in pairs of two, we identified USNM PAL 706595 as a left molar because it shows the typical wear on the anterior side *Desmostylus* teeth that are partially worn. The lingual sides of cusps are slightly shifted to posterior (see figure 2; see also Inuzuka [5]). The total length of the tooth row is 63.08 mm, and the width is 36.62 mm. The cusps were not worn and have no roots; therefore, this tooth had not yet erupted. Among its major cusps, six cusps are almost the same height, but one major cusp is a little smaller than others (M in figure 3). The number of major cusps is greater than that of *Cornwallius sookensis* (five for B.C.Prov.MUS 486, B.C.Prov.MUS 491, USNM V 181174; figure 4*a*,*b*). Compared to *Ounalashkastylus*, the crown height of USNM PAL 706595 is higher, and the cusps are thicker (figure 4*a*,*c*). The holotype specimen of *Desmostylus hesperus* (YPM 1395) only shows fragmentary teeth, but YPM 1395 is also a lower, unworn molar with no roots, making it comparable with USNM PAL 706595. The maximum crown height of USNM PAL 706595 is 43.20 mm. On the other hand, the maximum crown height of YPM 1395 is 48.18 mm. The referred specimen of *D. hesperus* (GSJ-F7745) has two m1s in the lower jaw. Compared to m1s of *D. hesperus*, the length and width (L: 28.25 mm, W: 43.14 mm) are clearly smaller than USNM PAL 706595. The type specimen of *Desmostylus japonicus* (NMNS PV-5600) also has lower molars (m1) of *D. japonicus* (L: 30.56 mm, W: 46.48 mm) are also clearly smaller than USNM PAL 706595. As a putative m2, USNM PAL 706595 is almost the same length and width as m2 of *D. japonicus* (estimated L: 60.06 mm, estimated W: 39.90 mm) and *D. hesperus* (L: 66 mm, W:45 mm from Ijiri & Kamei [40]). Based on these comparisons, we proposed that USNM PAL 706595 is likely an m2. We note, however, that the crown height of this specimen is shorter than that of *D. japonicus* (71.96 mm) and comparable specimens of *D. hesperus*.

USNM PAL 706595 is mostly unworn tooth, but we can observe and measure the enamel and dentin from ventral view. USNM PAL 706595 has very thick enamel (6.7 mm), especially compared to *Seuku*, *Behemotops*, *Ashoroa* and paleoparadoxiids. This characteristic is similar to *D. japonicus* and *D. hesperus*.

The line of left cusps of USNM PAL 706595 is slightly off to the caudal side, but they are oriented in a straight line along the left column. The line of right cusps is slightly tilted to the caudal side (figure 3). In each cusp, the columns of entoconid and protoconid are straight, but the metaconid is slightly inclined rostrally, and other cusps slightly inclined caudally. Compared to the m2 of *D. japonicus* (NMNS PV-5600) and *D. hesperus* (GSJ-F7745), for USNM PAL 706595 the inclination of its columns are more strongly curved. The arrangements of major cusps are consistent with typical characteristics of *Desmostylus* [5].

USNM PAL 706595 has many cuspules surrounding its major columns (figure 3). There are large cuspules (H: 14–15 mm) on the caudal side of the teeth. In *D. hesperus* (YPM 1395 and GSJ-F7745), there are no cuspules around the major cusps. In some referred specimens of *Desmostylus* teeth from the middle Miocene Temblor Formation of California, there are tiny cuspules around the major cusps. For example, USNM PAL V 206257, ml of *D. hesperus*, has a tiny cuspule (cuspule height: 7.8 mm, main cusp height: 41.07 mm) on its rostral side; and USNM PAL V 206252, M2 of *D. hesperus*, also has a tiny cuspule (cuspule height: 11.7 mm, main cusp height: 39.46 mm) on the buccal side. *Desmostylus* teeth with cuspules are not solely represented by *Desmostylus* teeth from California. USNM PAL V 23637, a fragmentary tooth of *Desmostylus* sp. from the middle Miocene Aijiri Formation of Miyagi, Japan, also has a small cuspule (cuspule height: 7.18 mm, main cusp height: 55.28 mm). Thus, cuspules on *Desmostylus* teeth are not unusual features for *Desmostylus* teeth (see electronic supplementary material, table S1). Compared with other *Desmostylus* teeth with cuspules, USNM PAL 706595 has larger and more numerous cuspules. In other desmostylids, such as *Cornwallius* and *Ounalashkastylus*, there are many large and conspicuous cuspules around the main cusps (*Cornwallius*: B.C.Prov.MUS 486, B.C.Prov.MUS 491, USNM PAL V 181740; *Ounalashkastylus*: MOTA 2004.009.03, MOTA 2004.0009.04, MOTA 2004.0009.05). The cuspules that surround molar teeth are characteristics that can be seen widely in desmostylids, although we note some specimens, such as USNM PAL V 206257, have more cuspules than typically seen in *Desmostylus*.

## 4. Discussion

Currently, there are three valid species of *Desmostylus*: *Desmostylus hesperus* Marsh [41], *D. japonicus* and *D. coalingensis* based on ontogenetic investigation and phylogenetic analysis [3,42]. Inuzuka *et al*. [5] and Kohno [15] suggested that the stratigraphic range of each *Desmostylus* species does not overlap, although this suggestion requires refinement with the available record of referred material, including specimens such as USNM PAL 706595. The type specimen of the oldest species, *D. japonicus*, was collected from the Akeyo Formation, Mizunami Group, Gifu Prefecture, Japan. Diatoms associated with the specimen place its geologic age at nearly 17 Ma [15]. Inuzuka *et al*. [5] assigned USNM PAL 181744, 187310 and 214741 from the Nye Formation to *D. japonicus* without explanation; currently, the Nye Formation ranges between 27.4 and 20.7 Ma [22], and thus a Chattian age cannot be excluded for some or all of these specimens, pending the resolution of their stratigraphic and geochronologic data.

Regardless of the stratigraphic overlap between species of *Desmostylus*, there remain open questions about the temporal and spatial co-occurrences of desmostylids and paleoparadoxiids in the North Pacific [43]. Data from the early Miocene of western North America demonstrate such overlap for *Desmostylus* and paleoparadoxiids (at around 20 Ma, based on this study and [31]). However, data from Japan only show the presence of paleoparadoxiids at this time and older [44,45]; the oldest known desmostylids from Japan are reported from 18 Ma rocks from Fukushima, Japan [13,14,24]. Thus, paleoparadoxiids appear nearly contemporaneously on both sides of the North Pacific around 20 Ma [27,44], while the appearance of *Desmostylus* in the western Pacific (around 19 Ma [13]) is later than the eastern side (no older than 23.8 Ma, this study). Before this time, in the Oligocene, desmostylian assemblages were more endemic in composition, with three genera from Japan and six from western North America, and only one genus (*Behemotops* [1,7]) shared between them.

It is unclear whether *Desmostylus* ranges into the Oligocene, but we cannot exclude this possibility based on specimens from the Nye Formation of Oregon. There are desmostylid occurrences in the Oligocene from both the western and eastern North Pacific [1,14,43–45], but there are no desmostylodont desmostylids from rocks in the western side of the Pacific; desmostylodont desmostylids first appeared on the eastern side of the Pacific, and expanded to the western side after the beginning of the Miocene.

USNM PAL 706595 has many cuspules around the tooth crown. While this feature seems rare (or rarely reported) among *Desmostylus* teeth (*D. japonicus* and *D. hesperus* from both Pacific coasts), we observed cuspules in 32% of specimens from reported desmostylid teeth, across three genera and four species (electronic supplementary material, table S1). Notably, all cuspule sizes in *Desmostylus* specimens that we observed were smaller than those in USNM PAL 706595. On the other hand, other desmostylids, such as *Cornwallius* and *Ounalashkastylus*, have more cuspules than *Desmostylus* (electronic supplementary material, table S1). While USNM PAL 706595 shows diagnostic features of *Desmostylus*, the presence of cuspules is a trait shared with basal branching desmostylians, such as *Behemotops* from the Oligocene. We hesitate to infer whether this trait represents species-level distinctions in *Desmostylus* from known or undescribed species.

## 5. Conclusion

In this study, we described a lower molar (USNM PAL 706595) diagnostic of *Desmostylus* from the earliest Miocene (Aquitanian) Skooner Gulch Formation of Mendocino County, California, USA. We used a combination of archival museum records and comparative descriptions in the literature to relocate its source locality, and its stratigraphic relevance following several decades of subsequent fossil discoveries from the Skooner Gulch Formation. It is likely that *Desmostylus* appeared before the earliest Miocene. USNM PAL 706595 demonstrates the specialized columnar teeth morphology of this genus persisted for more than 15 million years. We also suggest that future studies should revise the typology of *Desmostylus*, which is fragmentary and not as diagnostic as referred specimens.

## Ethics

USNM PAL 706595 was collected under the authority of the United States Geological Survey in the late twentieth century, and it is deposited in the collections at USNM.

## Data Availability

All the data are included in the article and electronic supplementary material. The original data are published at https://doi.org/10.5281/zenodo.7484877 [46]. All three-dimensional models we used in the paper are published at https://doi.org/10.17602/M2/M514952 [30], https://doi.org/10.17602/M2/M514948 [28], https://doi.org/10.17602/M2/M514928 [29]. The data are provided in electronic supplementary material [26].
